# Oar-miR-432 Regulates Fat Differentiation and Promotes the Expression of *BMP2* in Ovine Preadipocytes

**DOI:** 10.3389/fgene.2022.844747

**Published:** 2022-04-26

**Authors:** Meilin Jin, Xiaojuan Fei, Taotao Li, Zengkui Lu, Mingxing Chu, Ran Di, Xiaoyun He, Xiangyu Wang, Yuqing Wang, Zehu Yuan, Kai Quan, Huihua Wang, Caihong Wei

**Affiliations:** ^1^ Institute of Animal Sciences, Chinese Academy of Agricultural Sciences, Beijing, China; ^2^ College of Animal Science and Technology, China Agricultural University, Beijing, China; ^3^ Lanzhou Institute of Husbandry and Pharmaceutical Sciences, Chinese Academy of Agricultural Sciences, Lanzhou, China; ^4^ College of Animal Science and Technology, Henan University of Science and Technology, Luoyang, China; ^5^ College of Animal Science and Technology, Yangzhou University, Yangzhou, China; ^6^ College of Animal Science and Technology, Henan University of Animal Husbandry and Economy, Zhengzhou, China

**Keywords:** fat deposition, oar-miR-432, BMP2, preadipocytes, sheep

## Abstract

The fat tail is a unique characteristic of sheep that represents energy reserves and is a complex adaptative mechanism of fat-tailed sheep to environmental stress. MicroRNA plays a significant role as regulators at the posttranscriptional level, but no studies have explained the molecular mechanisms of miRNA which regulate fat deposition in sheep tails. In this study, mRNA and miRNA analysis examined tail fat tissue from three Hu fat-tailed and three Tibetan thin-tailed sheep. After aligning to the reference sequences, 2,108 differentially expressed genes and 105 differential expression miRNAs were identified, including 1,247 up- and 861 downregulated genes and 43 up- and 62 downregulated miRNAs. Among these differentially expressed miRNAs, oar-miR-432 was one of the most downregulated miRNAs between Hu sheep and Tibetan sheep, and 712 genes were predicted to be targeted by oar-miR-432, 80 of which overlapped with DEGs. The Gene Ontology analysis on these genes showed that *BMP2*, *LEP*, *GRK5*, *BMP7*, and *RORC* were enriched in fat cell differentiation terms. The genes for *BMP2* targeted by oar-miR-432 were examined using dual-luciferase assay. The oar-miR-432 mimic transfected into preadipocytes resulted in increased expression of *BMP2*. The marker gene *PPAR-γ* of fat differentiation had a lower expression than the negative control on days 0, 2, and 4 after induced differentiation. The decrease in the number of lipids in the oar-miR-432 mimic group detected by oil red O stain was also less than that in the negative control. This is the first study to reveal the fat mechanisms by which oar-miR-432 inhibits fat differentiation and promotes the expression of *BMP2* in sheep tails.

## Introduction

Sheep, as one of the first domesticated plant-eating animals, can be traced back to the Neolithic period about 11,000 years ago (Lawson Handley et al., 2007), especially in the Near East and Middle East based on existing archaeological evidence (Naval-Sanchez et al., 2018). To adapt to different environments, thin-tailed sheep evolved into fat-tailed sheep approximately 5,000 years ago, and the fat deposition in the tail or buttocks of sheep has become a desirable characteristic after domestication (Lv et al., 2015). Fat-tailed sheep makeup a quarter of the world’s sheep breeds (Mwacharo et al., 2017). In China, there are more than 98 indigenous sheep breeds, of which 80% are fat-tailed sheep. Based on the tail type, five types of the sheep can be defined (Mastrangelo et al., 2019).

With the improvement in people’s living standard, mutton consumption has been increasing, but the utilization rate of fat in sheep is less. The tail fat deposits are denser than those in other regions of the body (Bakhtiarizadeh et al., 2019). Excessive fat deposition affects the feed conversion rate of sheep, which increases the cost of farmers’ breeding. In production, a large amount of tail fat is directly discarded as waste, so it is necessary to conduct genetic improvement through modern breeding to reduce fat deposition and improve the feed conversion rate of sheep.

Given the importance of sheep tail in breeding, and its economic value, the regulatory mechanisms of fat deposition in the sheep tail are significant to understand. Until now, studies of fat deposition in sheep tail have mainly been concentrated on various genomic approaches, and during the proliferation and differentiation of preadipocytes, previous studies that indicated genes and noncoding RNAs were involved (Bakhtiarizadeh and Salami, 2019).

At the posttranscriptional level, microRNAs (miRNAs) are a significant class of gene regulators (Krol et al., 2010), where the seed region of miRNA combines with the 3′-UTR of genes to induce degradation or inhibit target gene translation (Bartel, 2009). In many organisms, miRNAs have been identified and are essential for cell proliferation and differentiation (Ge et al., 2019). An example is myoblast proliferation, where miR-10b-5p normally decreases steadily, but during myoblast differentiation, it increases significantly (Ge et al., 2019). However, no study has investigated the biological mechanisms of fat deposition of sheep tail by combined miRNA-seq and mRNA-seq.

In this study, miRNA-seq and mRNA-seq were used to identify potential miRNAs and mRNA related to sheep fat deposition in Hu fat-tailed and Tibetan thin-tailed sheep breeds. The oar-miR-432 associated with fat synthesis was identified, which is also one of the most downregulated miRNAs (Hao et al., 2021). The TargetScan, miRanda, and RNAhybrid packages of software were used to predict the potential target genes of oar-miR-432 (John et al., 2004; Krüger and Rehmsmeier, 2006; Agarwal et al., 2015) and putative target genes that overlapped with differentially expressed genes (DEGs). The effects of oar-miR-432 on the differentiation of ovine preadipocytes and its target genes were analyzed. The aim of this study was to understand the underlying molecular mechanisms of fat deposition in sheep tails, which would offer a basis for the genetic improvement of fat-tailed sheep breed.

## Materials and Methods

### Identified DEGs and DE miRNAs Identified and GO Enrichment Analysis

Six 18-month-old rams with similar weights in June, consisting of three pure-bred fat tailed Hu sheep from Yongdeng, Gansu, and three pure-bred thin tailed Tibetan sheep from Yushu, Qinghai, were used in this study. Tail fat from the rams was collected and washed with 0.9% NaCl immediately and then was frozen in liquid nitrogen. RNA was extracted by TRIzol (Invitrogen, Carlsbad, CA, United States) and sequenced by BGI (Shenzhen, China). The mRNA-seq and miRNA-seq data were mapped to *Ovis_aries* (Oar_v3.1), and related DEGs and differential expression miRNAs (DE miRNAs) were identified. Putative target mRNAs of oar-miR-432 were predicted by three different predictive software (RNAhybrid, TargetScan, and miRanda algorithm) (John et al., 2004; Krüger and Rehmsmeier, 2006; Agarwal et al., 2015). The data were uploaded in the SRA database (https://www.ncbi.nlm.nih.gov/sra) as PRJNA792697 and PRJNA777369. The significant enrichment of Gene Ontology (GO) functional terms with overlapped genes that showed differential expression was analyzed, which covered the three domains of cellular component, molecular function, and biological process (Chen et al., 2020).

### Plasmid Vector Construction and Transfection

The oar-miR-432 seed motif was mutated at the 3^’−^UTR site of *BMP2*. The primers were 5′-gtt​taa​aca​cat​ttA​GAA​CCC​tgt​taa​acc​cat​ttc​aga​caa-3′ for mut1 and 5′-aat​ggg​ttt​aac​aGG​GTT​CTa​aat​gtg​ttt​aaa​cac​ata​acc​tta​gaa-3′ for mut2. The experimental groups were set up as follows: BMP2-WT + mimics NC, BMP2-WT + oar-miR-432, BMP2-MUT + mimics NC, and BMP2-MUT + oar-miR-432. The HEK293T cells were resuscitated and cultured in complete medium in 24-well plates in an incubator at 5% CO_2_ and 37°C. When the cells grew to 60% or 70% confluence, they were cultured in Opti-medium (Gibco, United States) in quadruplicate, which contained 100 ng of the final construct and 20 nM of oar-miR-432 mimic (Trajkovski et al., 2012), with complete medium changed after 4–6 h. The cells were collected after transfection after 48 h. The Dual-Luciferase Reporter Assay System (Promega) was used for analysis.

### Isolation of Preadipocytes From the Adipose Tissue of Fat-Tailed Sheep and Transfection of the Oar-miR-432 Mimic

The preadipocytes were isolated from the tail fat of a 70-day-old Hu sheep fetus by collagenase digestion (Li et al., 2020) and cultured *in vitro* in a complete medium made up of 90% DMEM +5% fetal bovine serum (FBS) with 1% penicillin–streptomycin (PS) for 2 days at 37°C until the cells were almost adherent to the wall. The cells were subcultured to 6-well plates and cultured with 1000ul Opti-medium and 500 ng oar-miR-432 mimic in triplicate for 4–6 h before Opti-medium was replaced with a new complete medium. When the cells showed contact inhibition, the complete medium was replaced with the induction differentiation medium consisting of 90% DMEM +5% FBS +1% PS + 0.5 mM isobutyl methylxanthine +10 mg/ml insulin +1 uM dexamethasone and cultured for 2 days, which set as the first day (Li et al., 2020). Finally, the maintenance differentiation medium of 90% DMEM +5% FBS +1% PS +10 mg/ml insulin was used to culture for a further 2 days. The cells were collected from the oar-miR-432 mimic and negative control (NC) at days 0, 2, and 4 to extract RNA and protein. The cells differentiated for 4 days were stained with oil red O solution (Solarbio, China).

### Real-Time Quantitative Polymerase Chain Reaction and Western Blot Analysis

TRIzol was used to extract total RNA. Reverse transcription and RT-qPCR reaction were descripted by Jin et al. (2020) with β-actin as the housekeeping gene. The stem-loop method was used to synthesize cDNA from miRNAs and miRNA Design V1.01 (https://www.vazyme.com/) was used to design the primers. The RT-qPCR reaction was conducted as previously described (Vazyme, China) with 5s as the housekeeping gene. Three biological replicates and triplicate technical replicates for each breed were collected. All experimental data were analyzed by using equation 2^−ΔΔCt^. The primer information is listed in Supplementary Table S1. The ANOVA program in SPSS version 19.0 was used for statistical analysis (Jin et al., 2020) and considered statistically significant at *p-value < 0.05*. GraphPad Prism software (San Diego, CA, United States) was used to draw plots.

The cells were extracted and 1 ml pre-cooling RIPA lysis buffer containing 1 mM PMSF was added to obtain the total protein, the concentration of which was measured with the bicinchonininc acid method (Beyotime, China). The proteins were separated on 10% SDS-PAGE (Epizyme, China) with 120 V for 90 min and transferred onto a PVDF membrane (Millipore, United States) at 350 mA for 40 min. The membrane was sealed for 5 min at room temperature with quick sealing fluid (Lablead, China) and washed thrice with TBST (Solarbio, China). The proteins were detected with rabbit monoclonal anti-β-tubulin (Proteintech, United States) and rabbit monoclonal BMP2(Proteintech, United States) and the secondary antibody (Proteintech, United States). The reaction band was developed by using enhanced chemiluminescence (Epizyme, China), and an image of the membrane was recorded with a JP-K600 imaging system (JiaPeng, China).

## Results

### DEG and DE miRNA Analysis

The BGISEQ-500 platform was used to conduct sequencing of six mRNA and miRNA libraries. Clean reads aligned on the sheep reference genome of Oar_v3.1. DESeq2 were used to analyze DEGs and DE miRNAs between fat-tailed and thin-tailed sheep. A total of 2,108 genes with the false discovery rate (FDR) *≤ 0.01* and | Fragments per kilobase of exon per million reads mapped (FRKM)|≥1.5 were identified as DEGs, obtaining 1,247 upregulated genes and 861 downregulated genes in the two sheep breeds. There were 105 DE miRNAs, 43 of which were upregulated and 62 were downregulated, with FDR *≤ 0.01* and a |FRKM|≥1.5.

### Culture and Identification of Preadipocytes

**FIGURE 1:**
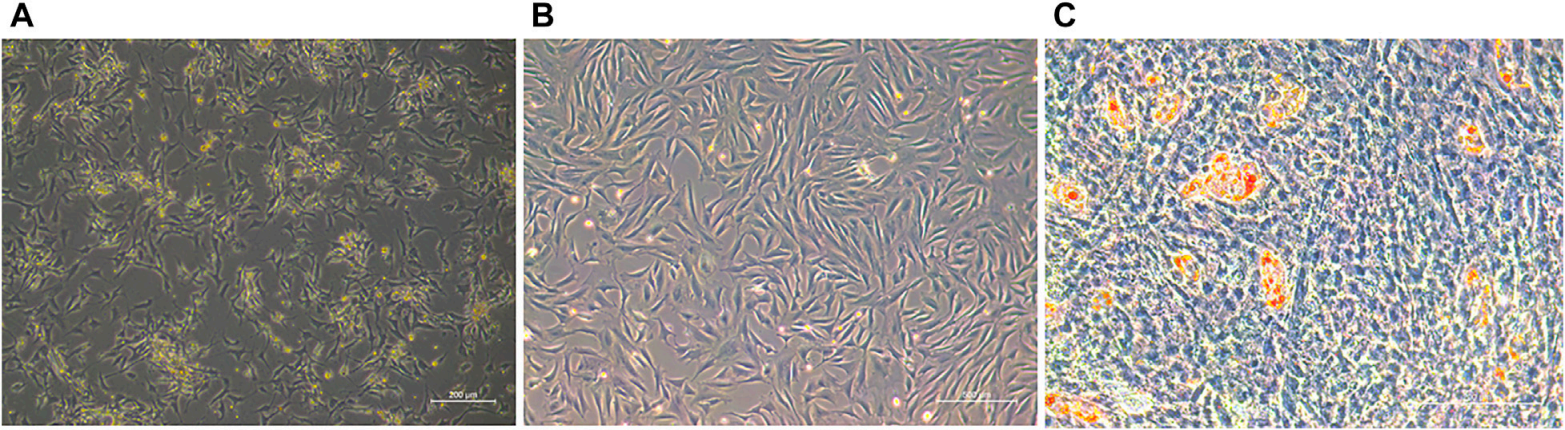
**(A)** Primary ovine preadipocytes (36 h). **(B)** Preadipocytes after three passages. **(C)** Adipocytes of oil red O staining.

The embryonic day 70 tail adipose tissues were collected and primary preadipocytes were cultured *in vitro* by collagenase digestion. These were mostly arranged in a fusiform shape (Figures 1A,B). When the cells reached a certain point, they stopped growing with contact inhibition and began to differentiate, and small lipid droplets appeared in the cell and accumulated into larger droplets, indicating that the cells were able to differentiate (Figure 1C).

### Effect of Oar-miR-432 on Inducing Differentiation of Preadipocytes

**FIGURE 2:**
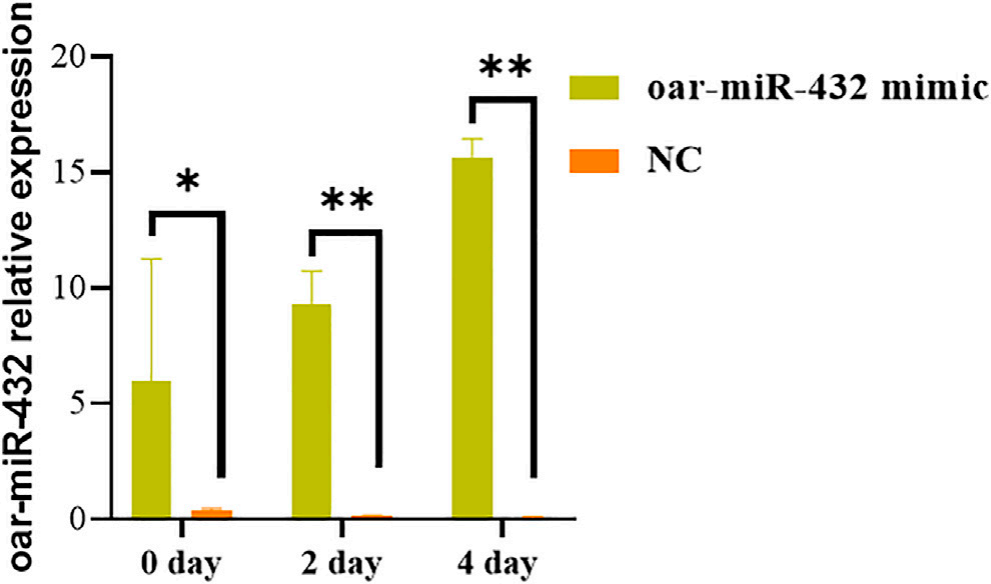
Relative expression levels of oar-miR-432 when oar-miR-432 mimic and NC were transfected into sheep preadipocytes.

The oar-miR-432 mimic and NC were transfected into preadipocytes with Opti-medium and then cultured with a new complete medium after 4–6 h. As contact inhibition was observed in the cells, the complete medium was replaced with the induction differentiation medium and cultured for 2 days, before culturing with the maintenance differentiation medium for further 2 days. The result indicated that the oar-miR-432 mimic in preadipocytes increased the expression level of oar-miR-432 on days 0, 2, and 4 (Figure 2). This result showed that the oar-miR-432 mimic was successfully transfected into the preadipocytes.

### Potential Target Genes of Oar-miR-432 and Overlapped With DEGs

**FIGURE 3:**
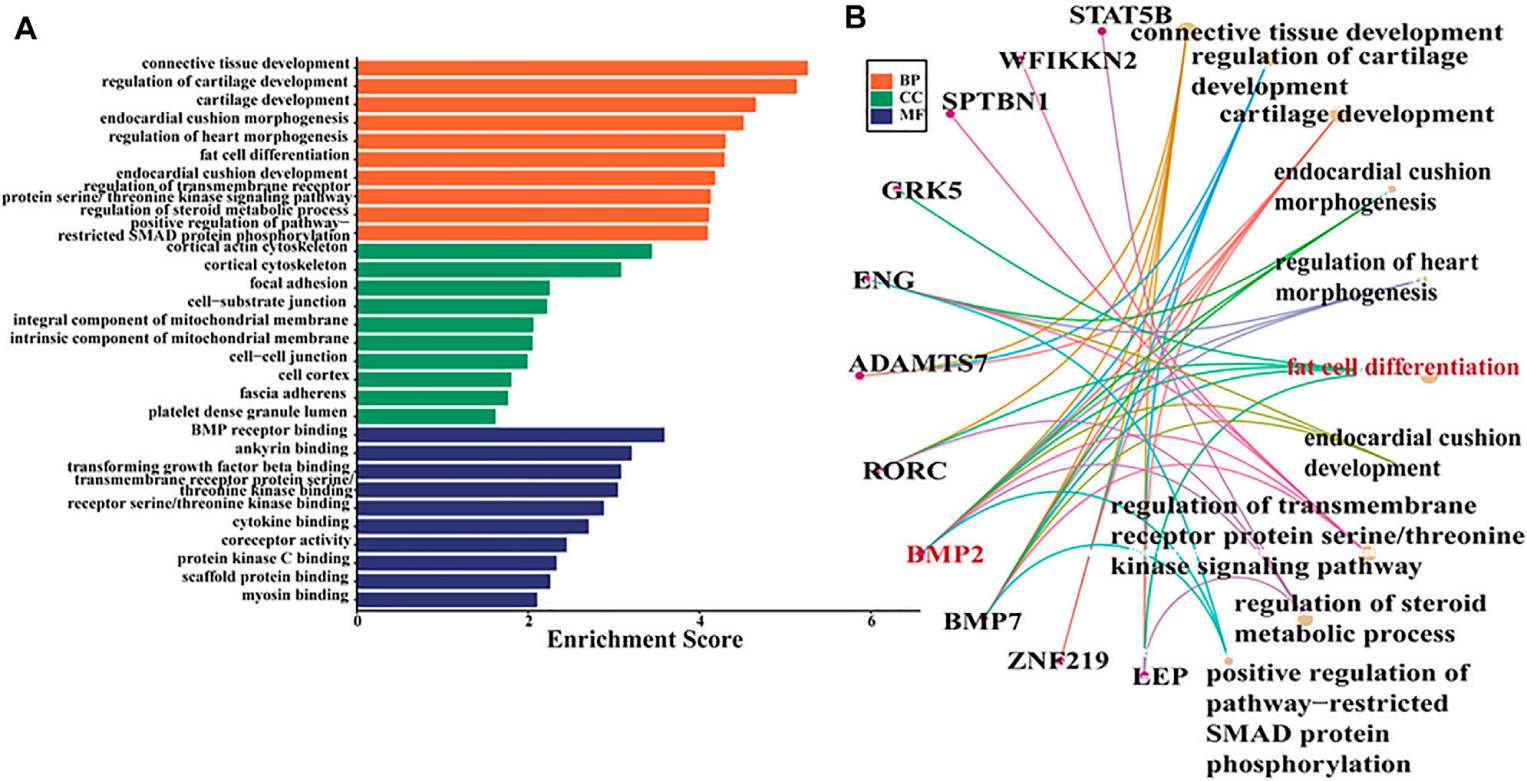
**(A)** GO term for overlapped genes between DEGs and target genes of oar-miR-432. **(B)** GO term associated with fat deposition in the biological process group.

In these studies, oar-miR-432 (FPKM = -2.66, *Q-value* = 1.86E-05) was downregulated between Hu and Tibetan sheep by miRNA-seq. Based on this result, the potential target genes of oar-miR-432 were predicted by the RNAhybrid, miRanda, and TargetScan software, where 712 genes were targeted, 80 of which overlapped with DEGs. Therefore, GO analysis was performed on these genes with the GO term demonstrating that most genes in fat cell differentiation were regulated (Figures 3A,B). Specifically, *BMP2* (FPKM = 1.80, *Q-value* = 7.32E-08), *LEP* (FPKM = 4.20, *Q-value* = 3.56E-20), *GRK5* (FPKM = -1.97, *Q-value* = 7.40E-07), *BMP7*(FPKM = -3.54, *Q-value* = 5.93E-06), and *RORC* (FPKM = 3.55, *Q-value* = 1.98E-08) were enriched by the positive regulation of fat cell differentiation. Target genes of oar-miR-432 were overlapped in differentially regulated mRNAs. In previous studies, 43 genes associated with fat tail development were identified by *Fst* and hapFLK. The genes *BMP2*, *HOXA11*, and *PPP1CC* potentially play significant roles in fat tail formation, where *BMP2* is the peak gene harbored in the largest region identified by hapFLK (Yuan et al., 2017). Selective scanning near the retrotransposition hotspot on chromosome 13 caused immobilization in domestic fat-tailed sheep and specifically affected the expression of *BMP2* (Pan et al., 2019; Lu et al., 2020). Based on these results, *BMP2* was selected for verification using the dual-luciferase reporter assay.

### Dual-Luciferase Reporter Assay to Verify Predicted Target Genes for Oar-miR-432

**FIGURE 4:**
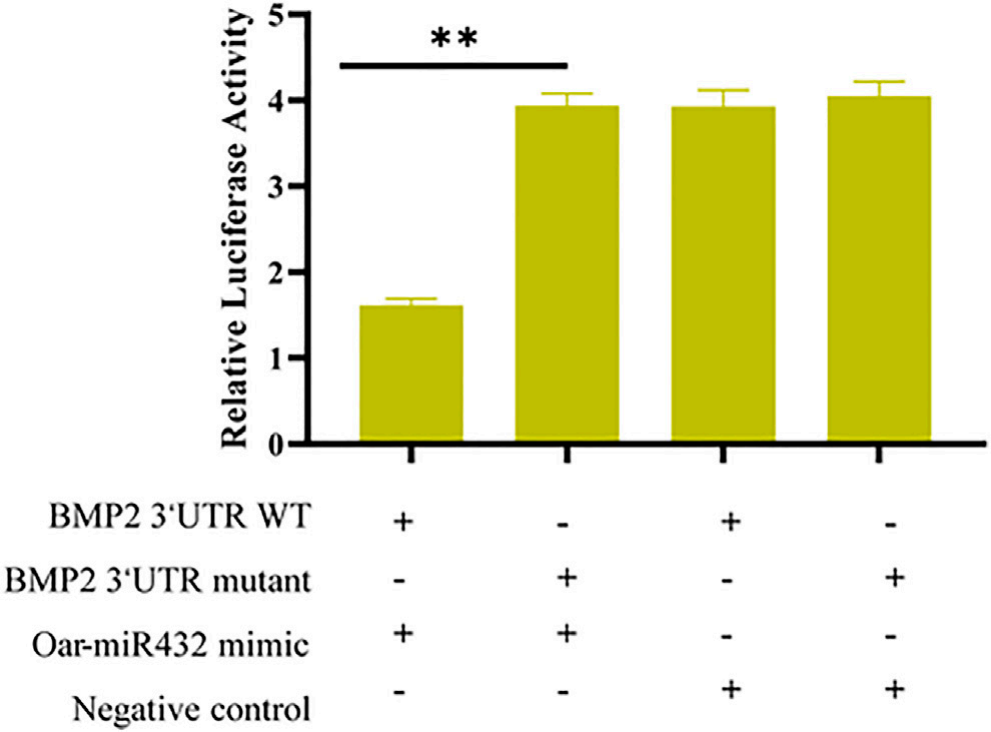
Dual-luciferase reporter assay to detect the predicted target gene *BMP2* of oar-miR-432.

Based on these results, to verify whether oar-miR-432 targeted *BMP2* directly, the 3′-UTR segment of *BMP2* was cloned into the psiCHECK2 luciferase reporter construct, which also had the predicted oar-miR-432 target site, or mutated seed sites (Supplementary Figure S1). The HEK293T cells were co-transfected with the reporter constructs with either oar-miR-432 or *BMP2* 3′-UTR wild type. The result indicated that the oar-miR-432 mimic containing a wild-type 3′-UTR reduced the reporter construct activity, but there were no changes when the luciferase reporter assays contained mutations in the seed sequences. This result demonstrated directly that *BMP2* was one of the target genes of oar-miR-432 directly (Figure 4).

### *BMP2* and *PPAR-γ* Expressions Are Regulated by Oar-miR-432

**FIGURE 5:**
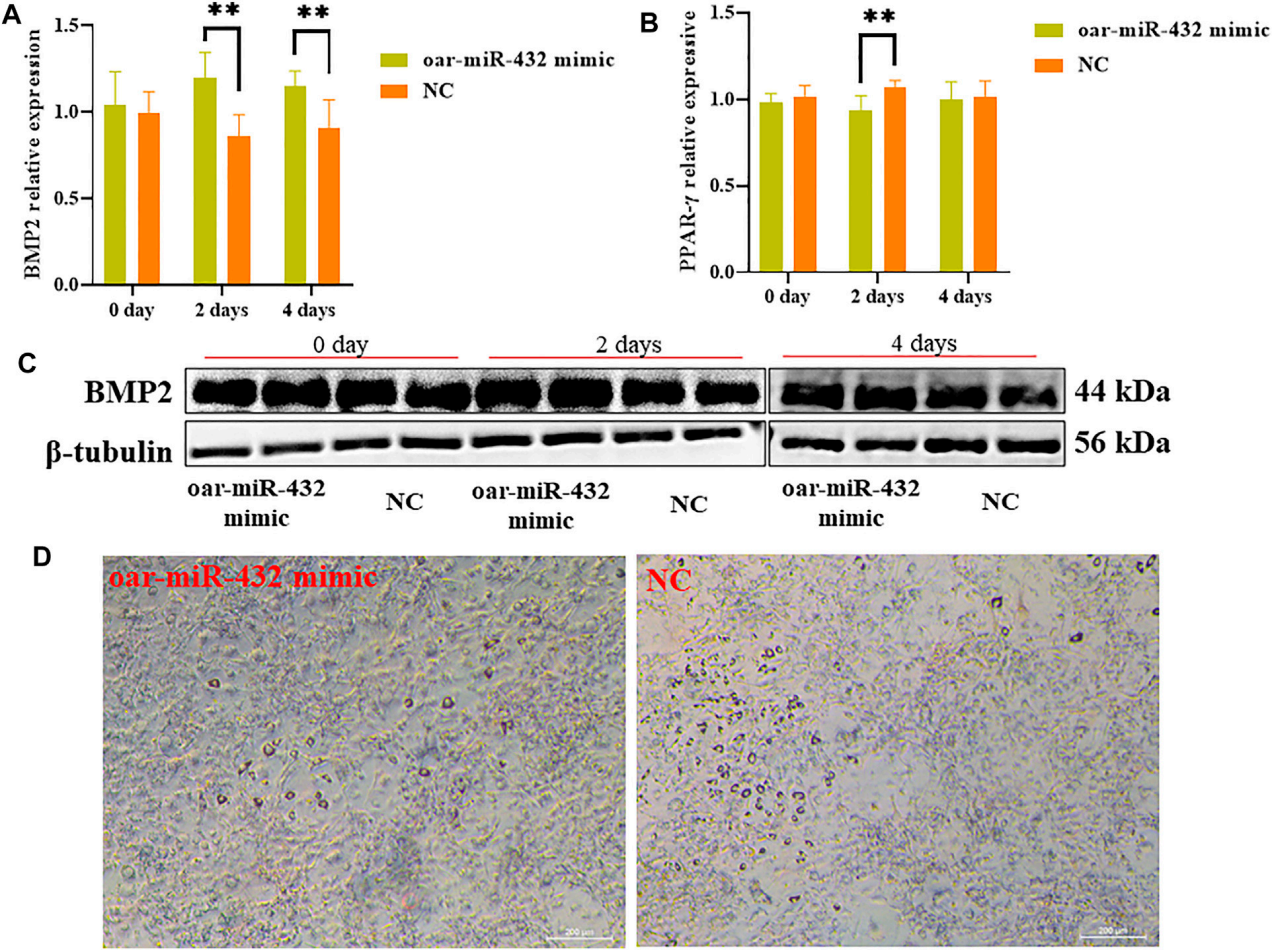
Relative expression of *BMP2* **(A)** and *PPAR-γ* **(B)**. **(C)** Oar-miR-432 mimic regulated the protein level expression of BMP2. **(D)** Oil red O staining when oar-miR-432 mimic in sheep preadipocytes after the maintenance differentiation medium.

The oar-miR-432 mimic was transfected into preadipocytes, and after induced differentiation increased, the mRNA levels of *BMP2* were compared with those of NC at days 2 and 4 (Figure 5A). The results of Western blot analysis also suggested that the oar-miR-432 mimic induced an increase in BMP2 expression of protein levels at days 2 and 4 (Figure 5C). The result demonstrated that oar-miR-432 overexpression promoted the expression of *BMP2* mRNA and protein. The expression level of the marker gene *PPAR-γ* was measured during adipogenesis, and oar-miR-432 resulted in downregulated expression levels after induction on days 0, 2, and 4, which was significantly lower than that of the NC at 2 days (*p < 0.01*) (Figure 5B).

Sheep preadipocytes on day 4 were stained with oil red O. Many small lipid droplets were stained red, and lipid rings were visible. The number of lipids drops in the oar-miR-432 mimic group was lesser than that of the NC (Figure 5D), which showed that fat deposition in sheep tail was decreased by oar-miR-432 overexpression.

## Discussion

Tail fat deposition in sheep has a complex genetic regulation mechanism, which is jointly determined by the environment and genes (Dong et al., 2020). In the summer and fall, the fat tail functions as stored energy, but during the cold winter and in harsh environments, it provides essential energy for sheep (Pan et al., 2019). In addition to the fat deposited in the skin and viscera of sheep like other mammals, the tail is also a major part of fat deposition, and like a camel’s hump, it can provide enough energy and heat for the body to grow in the harsh environment and during dry grass periods (Sbihi et al., 2013). Tail fat and its utilization is, therefore, an important part of fat metabolism research.

In this study, the relationship between *BMP2* and oar-miR-432 was first studied *in vitro*. The result suggested that oar-miR-432 can target *BMP2* directly. The oar-miR-432 mimic in preadipocytes led to the increased expression of *BMP2* and the decreased expression of *PPAR-γ*. These results indicated that the effect of oar-miR-432 was to inhibit fat differentiation during preadipocyte differentiation but promote *BMP2* expression. The regulatory mechanism of mRNA and miRNA in fat deposition remains poorly understood. Most studies merely identified important genes without subsequent validation *in vitro* or *in vivo*. For example, *BMP2* and *VRTN* were identified as potential candidates associated with fat-tailed sheep (Moioli et al., 2015) and some genes such as *BMP2*, *PDGFD*, *HOXA10*, *TBX12*, and *WDR92* were thought to be related to fat development in the fat-tailed sheep (Zhao et al., 2020). Genes such as *CDS2*, *PROKRI*, and *BMP2* under selection sweep were shown to be associated with lipid accumulation, and these studies revealed that *BMP2* was selected in the sheep tail associated with fat deposition (Baazaoui et al., 2021). The complex genetic factors associated with fat tail development still need further study, and the differences in fat deposition between Hu and Tibetan sheep is a valuable tool.

The effect of oar-miR-432 of ovine preadipocytes on the induced differentiation has not been reported previously. It has been shown that miR-432 inhibited milk fat synthesis in sheep mammary epithelial cells (Hao et al., 2021). During myoblast proliferation and differentiation, miR-432 was negatively regulated in pigs (Ma et al., 2017). The miR-432, also a regulator of *IGF2*, activates the related signaling pathway, and in bovine primary myoblasts, it combines with CircTTN to promote proliferation and differentiation (Wang et al., 2019). In Tibetan sheep, oar-miR-432 was expressed 2.66-times more than Hu sheep. When the oar-miR-432 mimic was transfected into preadipocytes and induced differentiation, *PPAR-γ* significantly decreased on day 2, which suggested that oar-miR-432 decreased fat deposition in sheep tails. In this study, the effect of oar-miR-432 in ovine preadipocytes was consistent with the miRNA-seq result that oar-miR-432 was downregulated in Hu sheep, which helped infer that miR-432 was an important negative regulator of fat deposition in sheep tails.

MiRNAs mainly repress gene expression by binding to mRNA (Chen et al., 2017), which form RNA-induced silencing complexes that lead to degradation or translation inhibition (Bushati and Cohen, 2007; Fabian et al., 2010; Chen et al., 2017; Stavast and Erkeland, 2019). However, after binding, some miRNAs were also found to directly promote the expression of genes (Ni and Leng, 2016; Chen et al., 2017), and mRNAs without caps and typical Poly (A) tails were more easily enhanced by miRNAs (Chen et al., 2017; Cui and Joo, 2019). It has been reported that the interaction between miRNA and mRNA is dynamic and the activation of miRNA-dependent mRNA translation depends on both conditional and cellular constraints (Ni and Leng, 2016). It was reported that under serum deprivation, miR-369 could switch from translation repression to activation (Buchan and Parker, 2007) and that miR-122, after combining with the sites in the 5′-UTR of HCV RNA, could positionally regulate the viral life cycle (Roberts et al., 2011). The specific induction of miR-1 during myogenesis allows it to efficiently enter the mitochondria, stimulating the translation of transcripts encoded by specific mitochondrial genomes (Zhang et al., 2014). During desiccation, Cgi-miR-365 combined with the 3′-UTR of *CgHSP90AAl* to promote *CgHSP90AAl* expression directly (Chen et al., 2017). Based on previous studies, miRNAs promoted the expression of genes, but the detailed mechanism is still largely unknown.

## Conclusion

In this study, the interaction between oar-miR-432 and *BMP2* was verified *in vitro*. Oar-miR-432 inhibits fat differentiation and promotes the expression of the target gene *BMP2* in ovine preadipocytes. The present results failed to demonstrate the exact mechanism of how the expression of *BMP2* was promoted by oar-miR-432. It was speculated that the *BMP2* mRNA was protected by oar-miR-432 from degradation, which might be vital in sheep fat-deposition metabolism. These results provide added information to help understand the miRNA-mediated adaptation mechanism in controlling sheep tail fat deposition.

## Data Availability

The datasets presented in this study can be found in online repositories. The names of the repository/repositories and accession number(s) can be found at: https://www.ncbi.nlm.nih.gov/, PRJNA792697 https://www.ncbi.nlm.nih.gov/, PRJNA777369.
