# Supplementary figures and images for: Oar-miR-432 Regulates Fat Differentiation and Promotes the Expression of BMP2 in Ovine Preadipocytes
Source: Front Genet. 2022 Apr 26;13:844747. doi: 10.3389/fgene.2022.844747 (PMC9087340; doi:10.3389/fgene.2022.844747)

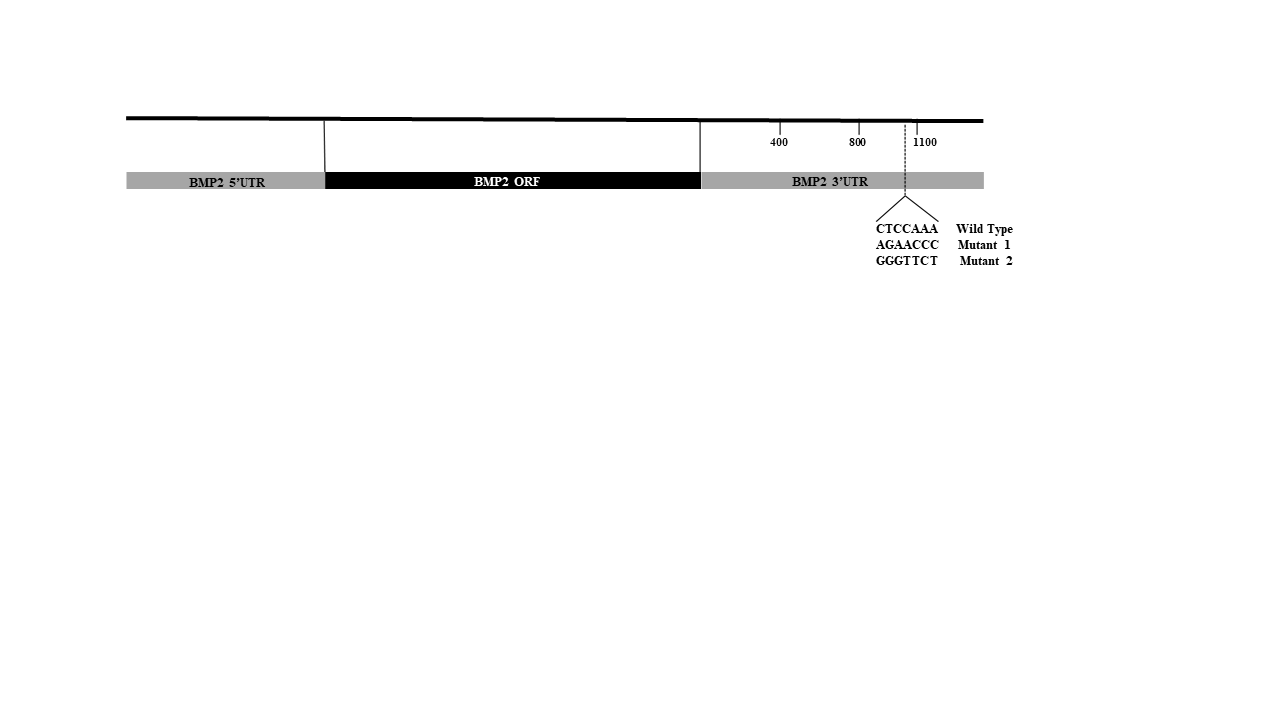

Supplement: Supplementary file 1 [file Image1.TIF]
